# Nomogram prediction model for pneumonia after valve replacement in patients with heart valve disease

**DOI:** 10.3389/fcvm.2026.1670003

**Published:** 2026-01-30

**Authors:** Yunhong Liu, Xujing Wang, Shuhui Wang, Yingjuan Cao

**Affiliations:** 1 Nursing Department, Qilu Hospital, Shandong University, Jinan, China; 2 Nursing Theory and Practice Innovation Research Center, Shandong University, Jinan, China; 3 Cardiac surgery Intensive Care Unit, Shandong Provincial Hospital Affiliated to Shandong First Medical University, Jinan, China; 4 Infection Control Department, Qilu Hospital of Shandong University, Jinan, China

**Keywords:** heart valve disease, hospital-acquired pneumonia, nomogram, postoperative recovery, pulmonary hypertension, valve replacement

## Abstract

**Background:**

Patients with heart valve disease (VHD) combined with pulmonary hypertension (PH) have a higher risk for hospital-acquired pneumonia. We aimed to analyze the risk factors and construct a valid nomogram model for predicting hospital-acquired pneumonia among those patients.

**Methods:**

Patients with VHD combined with PH who underwent heart valve replacement were collected. The perioperative risk factors for hospital-acquired pneumonia were analyzed by univariable and logistic regression, and then a nomogram prediction model was constructed and validated.

**Results:**

A total of 377 patients were included, and 81 cases developed postoperative hospital-acquired pneumonia, with an incidence of 21.49%. The results of multifactorial analysis showed that preoperative anemia, ASA score >grade III, duration of surgery ≥313 min, duration of endotracheal intubation ≥3 d, duration of indwelling gastric tube ≥1 d, and bioprosthetic valve usage were the risk factors for the occurrence of postoperative hospital-acquired pneumonia (*P* < 0.05). The model validation results showed that patients judged to be at high risk of hospital-acquired pneumonia are consistent with the actual situation, indicating that the model has good predictive efficacy.

**Conclusions:**

The constructed six-variable nomogram prediction model has satisfying efficacy in predicting hospital-acquired pneumonia after valve replacement in patients with VHD combined with PH. It is significant for early identification and future quality improvement to reduce the risk of hospital-acquired pneumonia.

## Background

Valvular heart disease (VHD) is a common heart disease worldwide. Previous surveys showed regional variations in the prevalence of VHD, with 2.5% in the United States, 11.3% in the United Kingdom, and 9.65% in the Chinese population (1, 2). The causes of VHD are complex, and the prolonged duration of the disease severely impairs patients' physical function and their quality of life (3). As the disease progresses, patients may also develop cardiac insufficiency and hemodynamic changes (4). Pulmonary hypertension (PH) is a common comorbidity of VHD, and its occurrence is closely related to valvular disease and heart failure (5–7). The study showed that 32.4% of patients with heart disease had comorbid PH (8). Combined PH exacerbates the impairment of cardiopulmonary function in patients with VHD, leading to a poor prognosis and raising the risk of death (9, 10).

Currently, the primary clinical treatment for VHD patients is heart valve replacement surgery (11, 12). Valve replacement is a complex cardiac procedure with a high risk of postoperative hospital-acquired pneumonia (13). Hospital-acquired pneumonia can further exacerbate patients' postoperative cardiopulmonary burden, leading to poor prognosis and even death (14). Previous studies have shown that the incidence of hospital-acquired pneumonia after valve replacement ranged from 11.4% to 33.33% (13, 15–18). Various risk factors contribute to its occurrence. It has been reported that PH is an independent risk factor for the development of postoperative hospital-acquired pneumonia, which indicates that patients with VHD combined with PH are at high risk of developing hospital-acquired pneumonia after valve replacement (16). Furthermore, studies indicated that postoperative pneumonia was significantly associated with perioperative factors such as the duration of surgery, blood cell transfusion, etc. (17).

The nomogram model is based on the regression model, which can integrate various risk factors, using graphical and visualization methods. It is suitable for individualized prediction of the probability of postoperative hospital-acquired pneumonia in patients with high clinical utility (19). Few studies have analyzed the risk factors and constructed nomogram models for predicting hospital-acquired pneumonia after valve replacement among patients with VHD combined with PH. For example, a previous study evaluated the predictive value of procalcitonin in ventilator-associated pneumonia after cardiac valve replacement but did not construct a prediction model (20). Therefore, this study will fill the research gap. The hypotheses are that a) the significant perioperative risk factors for hospital-acquired pneumonia after valve replacement in patients with VHD combined with PH can be identified; b) the constructed nomogram model has good validity for predicting hospital-acquired pneumonia.

## Methods

### Participants

The clinical data of 377 patients with VHD complicated by PH who were treated at a tertiary general hospital in Shandong Province from January 2022 to December 2023 were retrospectively collected. Inclusion criteria: (1) age ≥18 years old; (2) those who were diagnosed with heart valve disease combined with pulmonary hypertension, which was measured by transthoracic echocardiography (defined as pulmonary artery pressure plus central venous pressure >60 mm Hg) before the operation, and met the diagnostic criteria of the Chinese Guidelines for the Diagnosis and Treatment of Pulmonary Arterial Hypertension (2021 edition) (21); (3) those who underwent cardiac valve replacement treatment; (4) those who had complete medical records. Exclusion criteria: (1) people with congenital heart disease combined with pulmonary hypertension; (2) people with a postoperative combination of infections in other parts of the body; (3) people with other severe organic lesions, hearing, and mental disorders; (4) people with preoperative pulmonary infection.

### Data collection

The clinical data of participants were obtained from the hospital information system, laboratory information system, and the hospital infection surveillance system, including demographic, diagnostic, treatment data, and surgical information. The physicians and infection management experts reviewed all the infected cases and entered into the hospital infection surveillance system. Diagnostic specialists were blinded to other patients' data. The hospital-acquired pneumonia was diagnosed based on criteria by the Centers for Disease Control and Prevention. The study was approved by the hospital ethics committee (KYLL-2020-149). The need for patients informed written consent was waived by the hospital ethics committee.

### Statistical analysis

SPSS 22.0 and R 4.1.3 software were used for data analysis and nomogram model construction. Variables with normal distribution were expressed as mean ± standard deviation, and the *t*-test was used for intergroup comparison; the count data was expressed as rate or percentage, and the *χ*^2^-test was used for intergroup comparison; some continuous variables, including preoperative length of stay, duration of surgery, duration of the aortic block, duration of extracorporeal circulation, etc. were transformed into dichotomous variables according to the best cut-off value which was determined through Youden index in ROC curve analysis. The variables with statistically significant differences (*P* < 0.05) after univariate analyses were included in the multifactorial logistic regression analysis (forward-LR method). Independent risk factors for postoperative hospital-acquired pneumonia were analyzed based on the screened independent risk factors, and a nomogram model was drawn using R software and its related program package. The Bootstrap method (repeated sampling 1,000 times) was adopted to validate the model internally. The calibration curves, the (corrected) C-index, the Hosmer-Lemeshow test, the clinical decision curve, and the clinical impact curve were used to assess the predictive efficacy of the model. Differences were considered statistically significant at *P* < 0.05.

## Results

### Postoperative hospital-acquired pneumonia in patients with VHD combined with PH

Among 377 patients with VHD combined with PH receiving heart valve replacement surgery, there were 172 male and 205 female patients, with an average age of 57.21 ± 10.43 years, and a total of 81 patients developed postoperative hospital-acquired pneumonia, with an incidence of 21.49%.

### Univariate analysis of clinical data of patients with VHD combined with PH

Age, preoperative anemia, heart failure, valve material, preoperative hospital stay, left ventricular ejection fraction, American Society of Anesthesiologists (ASA) score, duration of operation, duration of aortic block, postoperative intensive care unit (ICU) stay, days of endotracheal intubation, days of non-invasive mechanical ventilation, days of central venous catheterization, days of indwelling gastric tube, duration of extracorporeal circulation, postoperative low cardiac output syndrome were statistically significant (*P* < 0.05) between groups, and the differences in New York Heart Association (NYHA) cardiac function classification, intraoperative blood transfusion, and body mass index between the two groups were not statistically significant, as shown in Table 1.

**Table 1:** Univariate analysis of clinical data of patients with VHD combined with PH.

Variables		Infected group	Uninfected group	*F*	*P* value
		(*n* = 81)	(*n* = 296)		
Gender	Male	33	139	0.991	0.319
	Female	48	157		
Age		60.04 ± 10.17	56.44 ± 10.38	−2.778	0.006^a^
BMI (kg/m^2^)		23.30 ± 4.14	24.04 ± 3.67	1.571	0.117
Smoking	Yes	11	66	2.973	0.085
	No	70	230		
Alcohol usage	Yes	12	62	1.515	0.218
	No	69	234		
Hypertension	Yes	21	81	0.067	0.796
	No	60	215		
Diabetes	Yes	7	22	0.131	0.717
	No	74	274		
Preoperative LOS (d)	<8	33	162	4.984	0.026^a^
	≥8	48	134		
Preoperative anemia	Yes	18	23	13.704	<0.001^a^
	No	63	273		
NYHA	I–II	17	85	1.925	0.165
	III–IV	64	211		
Heart failure	Yes	20	43	4.721	0.030^a^
	No	61	253		
Left ventricular ejection fraction (%)	<50	17	34	4.908	0.027^a^
	≥50	64	262		
ASA score	≤III	57	262	16.081	<0.001^a^
	>III	24	34		
Open surgery	Yes	81	289	0.870	0.351
	No	0	7		
Duration of surgery (min)	<313	35	204	18.114	<0.001^a^
	≥313	46	92		
Valve Materials	Mechanical valves	43	215	11.251	0.001^a^
	Biological valves	38	81		
Intraoperative blood transfusion	Yes	77	275	0.478	0.490
	No	4	21		
Duration of Aortic block (min)	<145	56	247	8.255	0.004^a^
	≥145	25	49		
Duration of Extracorporeal circulation (min)	<209	54	253	14.876	<0.001^a^
	≥209	27	43		
LOS in ICU (d)	<6	33	215	28.741	<0.001^a^
	≥6	48	81		
Number of days of tracheal intubation (d)	<3	49	280	66.559	<0.001^a^
	≥3	32	16		
Days of non-invasive mechanical ventilation (d)	<1	68	286	15.681	<0.001^a^
	≥1	13	10		
Days of central venous catheterization (d)	<11	39	210	14.740	<0.001^a^
	≥11	42	86		
Number of days of indwelling gastric tube (d)	<1	57	289	62.645	<0.001^a^
	≥1	24	7		
Postoperative low cardiac output syndrome	Yes	14	5	29.141	<0.001^a^
	No	67	291		

a represents *P* < 0.05.

BMI, body mass index; ICU, intensive care unit; NYHA, New York Heart Association; LOS, length of stay; ASA, American Society of Anesthesiologists.

The 16 variables showing statistical significance on univariate analysis were included as independent variables in multifactorial logistic regression (forward-LR method) analysis. The results showed that preoperative anemia, ASA score >grade III, operative time ≥313 mins, and days of tracheal intubation ≥3d, days of bioprosthetic valve and indwelling gastric tube ≥1d were independent risk factors for postoperative hospital-acquired pneumonia in patients with VHD combined with PH (*P* < 0.05), as shown in Table 2.

**Table 2:** Multifactor logistic regression of postoperative hospital-acquired pneumonia in patients with VHD combined with PH.

Variables	*β*	standard error	*Wald χ²*	*P* value	*OR*	95%*CI*
Preoperative anemia	0.860	0.412	4.359	0.037^a^	2.363	1.054–5.297
ASA score >III	0.777	0.355	4.778	0.029^a^	2.174	1.084–4.362
Duration of surgery ≥313 min	0.659	0.302	4.776	0.029^a^	1.933	1.070–3.491
Biological valves	0.862	0.301	8.224	0.004^a^	2.369	1.314–4.270
Number of days of indwelling gastric tube ≥1 d	1.768	0.561	9.922	0.002^a^	5.860	1.950–17.610
Number of days of tracheal intubation ≥3 d	1.211	0.438	7.653	0.006^a^	3.358	1.424–7.922

a represents *P* < 0.05.

ASA, American Society of Anesthesiologists; OR, odds ratio; 95% CI, 95% Confidence interval.

### Construction and validation of a nomogram model for the risk of postoperative hospital-acquired pneumonia in patients with VHD combined with PH

**Figure 1:**
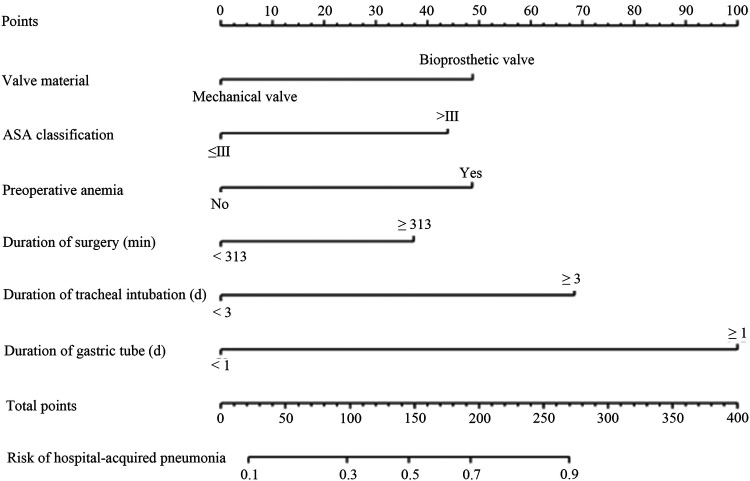
A nomogram predicting the risk of hospital-acquired pneumonia after valve replacement in patients with VHD combined with PH. Each variable's value was given a score on the point scale axis. A total core could be easily calculated by adding each single score, and by projecting the total score to the lower total point scale, we could estimate the probability of hospital-acquired pneumonia.

Based on the six independent risk factors obtained from the above regression analysis, a nomogram model for the risk of postoperative hospital-acquired pneumonia in patients with VHD combined with PH was drawn, as seen in Figure 1. The method of applying the nomogram was as follows: the six scores of valve materials, the ASA score, the preoperative anemia, the duration of the operation, the number of days of tracheal intubation, and the number of days of the gastric tube in place were summed to a total score, which represents different risks of infections. For example, a patient received a bioprosthetic valve, had an ASA score ≤ Grade III, presented with anemia preoperatively, underwent surgery lasting <313 min, required endotracheal intubation for <3 days, and had a nasogastric tube in place for <1 day. The six-item scores were 50 points, 0 points, 50 points, 0 points, and 0 points, resulting in a total score of 100 points. The corresponding hospital-acquired pneumonia risk probability on the total score axis is 30%.

**Figure 2:**
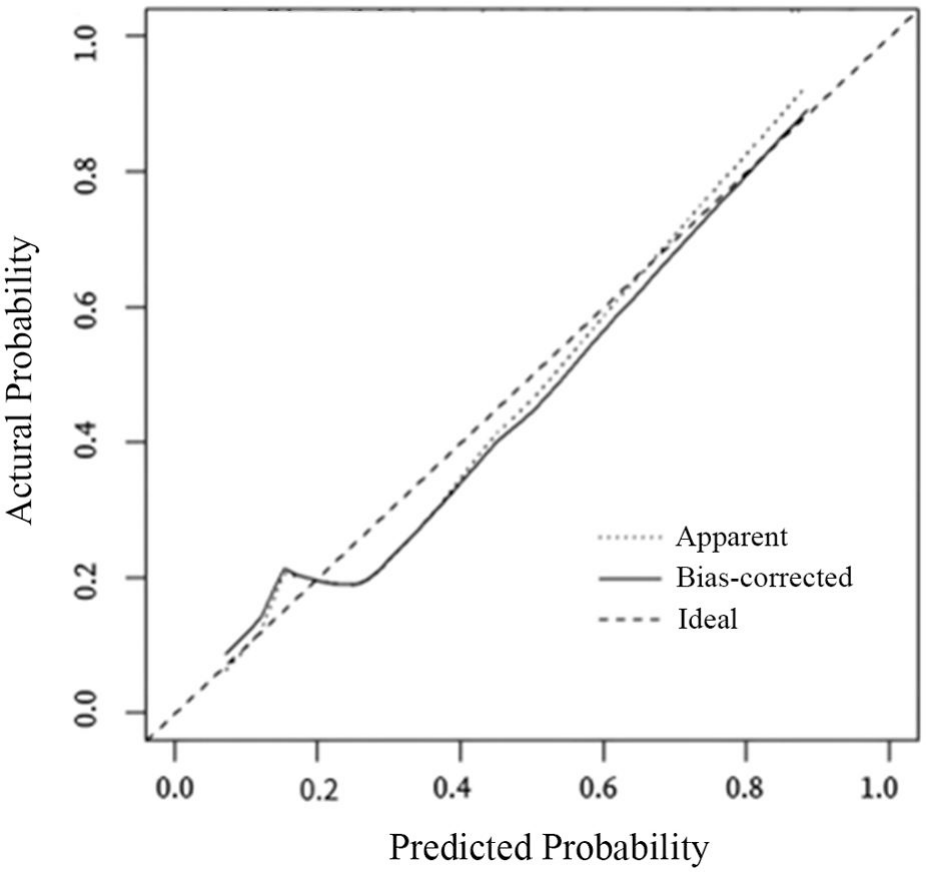
The calibration curves for the nomogram. The *x*-axis represents the nomogram-predicted probability, and the *y*-axis represents the actual probability of hospital-acquired pneumonia. A perfect prediction would correspond to the 45 ° dashed line. The dotted line represents the entire cohort (*n* = 377), and the solid line is bias-corrected by bootstrapping (B = 1,000 repetitions), indicating observed nomogram performance.

**Figure 3:**
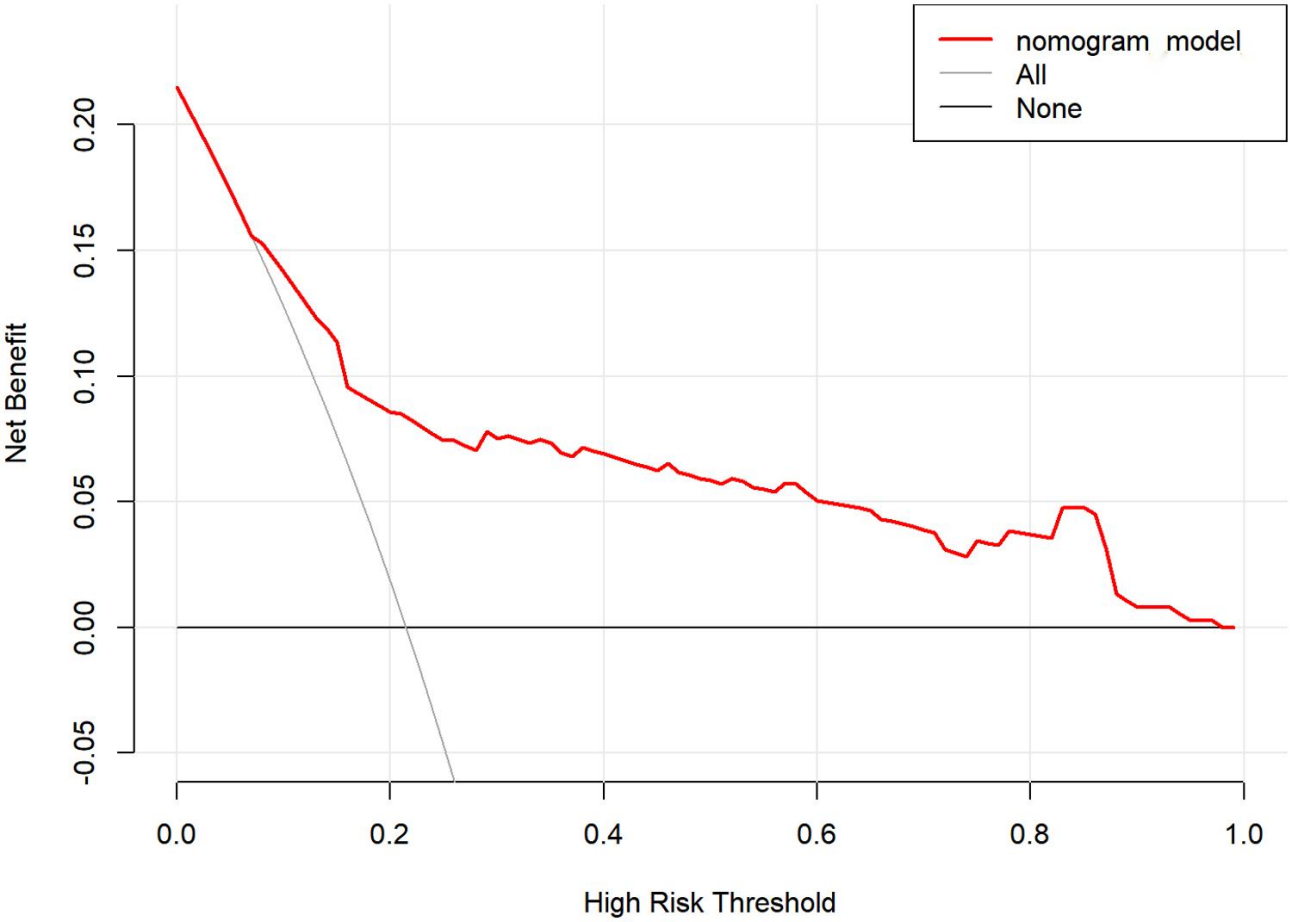
Decision curve of the nomogram model.

**Figure 4:**
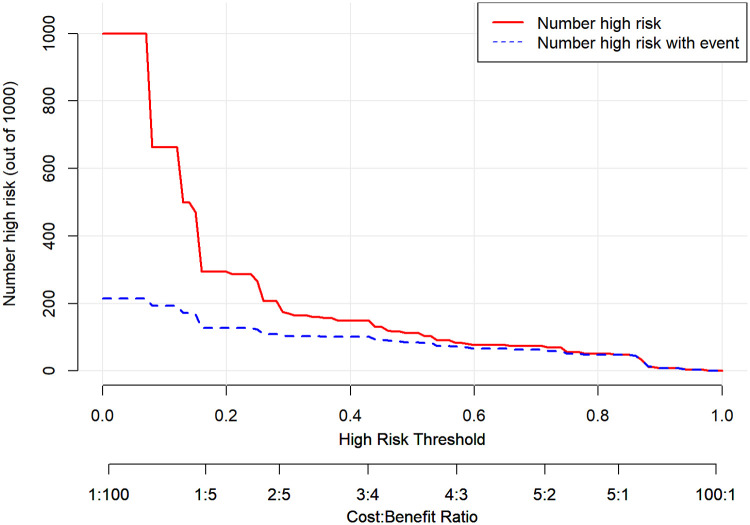
Clinical impact curve of the nomogram model.

The internal validation results of the model showed that the C-index was 0.782 (95% CI: 0.723–0.841), and the corrected C-index was 0.770; the Hosmer-Lemeshow test was *P* = 0.511; the calibrated curves of the nomogram model were close to the ideal curves, which indicated that the model had a reasonable degree of accuracy, as seen in Figure 2. The clinical decision curve showed that the model had a high net benefit value in the range of 10%–95%, with good clinical validity, see Figure 3. The clinical impact curve showed that when the threshold probability was greater than 0.5, the number of people judged by the model to be at high risk of postoperative hospital-acquired pneumonia was highly matched to the number of people who developed postoperative hospital-acquired pneumonia, with a high clinical validity rate, see Figure 4.

## Discussion

The present study identified six independent perioperative risk factors for postoperative hospital-acquired pneumonia in patients with VHD combined with PH. The nomogram model has good internal validation and significant clinical value in that it can help to identify high-risk patients during the early postoperative period. This study added clinical evidence and theoretical value to the occurrence of hospital-acquired pneumonia in patients with VHD combined with PH.

Valve replacement is the primary treatment method for patients with VHD combined with PH, which is a complex and invasive procedure with a high risk of postoperative hospital-acquired pneumonia. PH can cause lung injury in patients, which further increases the risk of postoperative hospital-acquired pneumonia in patients (22). The results of this study showed that 81 out of 377 surgical patients had postoperative hospital-acquired pneumonia, with an incidence of 21.49%, which was much higher than the 13.15% reported by Wei Runsheng et al. (15) but lower than the 33.3% reported by Zhao Ying et al. (18) This may be related to the differences in the population included in the study. Postoperative hospital-acquired pneumonia not only affects patients' lung function and increases postoperative cardiopulmonary burden but also increases treatment difficulties and risk of death (15). Therefore, it is important to construct a nomogram model for the risk of postoperative hospital-acquired pneumonia in patients with VHD combined with PH for early identification of high-risk patients.

The results of this study showed that ASA score >grade III, operative time ≥313 min, preoperative anemia, bioprosthetic valves, days of tracheal intubation ≥3d, and days of indwelling gastric tube ≥1d were the independent risk factors for postoperative hospital-acquired pneumonia after valve replacement surgery in patients with VHD combined with PH. A study reported by Morikane and other scholars showed that high ASA scores were strongly associated with postoperative infections after cardiac surgery, which is in line with the present study (23). The higher the ASA score grading, the poorer the patient's preoperative physical status and the higher the surgical and anesthetic risk. Patients with high ASA scores usually had poor surgical tolerance and higher risks of postoperative infections. The ASA score has been verified to have a good predictive effect on perioperative morbidity and mortality (24). Previous studies have shown that the duration of surgery is an independent risk factor for hospital-acquired infections in heart valve replacement, which is in line with the present study (16, 17). The risk of postoperative hospital-acquired pneumonia was higher in patients with a duration of surgery of ≥313 mins, and the reasons for this may be related to the long duration of surgery resulting in the prolonged exposure of the patient's organism to the external environment and increased exposure to infectious pathogens. A large number of studies reported that preoperative anemia was closely associated with adverse outcomes such as infection after cardiac surgery, supporting its inclusion as a predictive variable in risk prediction models (25–27). The reason may be related to the fact that preoperative anemia increases the intraoperative transfusion rate and the risk of postoperative complications, leading to an increased risk of postoperative infection in patients.

This study found that valve material was strongly associated with postoperative hospital-acquired pneumonia. Using bioprosthetic valves was an independent risk factor for postoperative hospital-acquired pneumonia in patients with VHD combined with PH. The previous studies showed that the incidence of adverse events such as postoperative endocarditis and reoperation was higher in patients with bioprosthetic valves than with mechanical valves (28, 29). Therefore, valve materials should be selected based on the patient's actual situation in clinical work, and mechanical valves may be a better choice for the population with VHD combined with PH. Zhao Ying et al. found that the extubating time of tracheal intubation was a risk factor for postoperative hospital-acquired pneumonia after valve replacement (18), and the present study similarly found that the number of tracheal intubation days ≥3d was independently associated with postoperative hospital-acquired pneumonia. Tracheal intubation leads to the communication between the patient's airway and the external environment, which makes it easier for pathogenic bacteria to invade the respiratory tract, and prolonged intubation is highly irritating to the organism, which is prone to cause respiratory mucosal damage and increased secretion, leading to an increased risk of postoperative hospital-acquired pneumonia in patients. Indwelling nasogastric tube is closely associated with respiratory complications, mostly related to intubation into the trachea by mistake and aspiration (30). In this study, we found that the risk of postoperative hospital-acquired pneumonia in patients with VHD combined with PH increased with prolongation of the postoperative indwelling gastric tubes. Therefore, intubation and nasogastric operation should be strictly observed in clinical work; accurate intubation, prevention of mis-aspiration, and early extubation are preferred.

In this study, we constructed a nomogram model for the risk of hospital-acquired pneumonia after valve replacement in patients with VHD combined with PH based on the multifactorial logistic regression analysis results. We used various methods to validate the predictive efficacy of the model, such as the C-index, the corrected C-index, the corrected curve, and the Hosmer-Lemeshow test. The results showed that the model had good predictive efficacy. In addition, the clinical validity of the model was verified in this study using clinical decision curves and clinical impact curves (31), and the results showed that the net benefit value of the model was high in the range of 10%–95%. The model determined that the number of high-risk individuals was a high match to the actual number of infected individuals when the threshold probability was greater than 0.5. Therefore, the model has good predictive efficacy and clinical validity.

There were some limitations in the present study. First, the samples were from a single center and were not validated in other hospitals. Additionally, our study converted certain continuous variables into binary variables based on the best cut-off values. This may result in information loss and potentially reduce model accuracy, although dichotomized continuous variables may have more clinical relevance. Last, the sample size did not support external validation of the model. Subsequent large-sample, multi-center studies are needed to validate the model further and make it truly applicable to clinical practice.

## Conclusions

In conclusion, this study found that ASA score > grade III, duration of operation ≥313 min, preoperative anemia, bioprosthetic valve, days of tracheal intubation ≥3d, and days of indwelling gastric tube ≥1d were independent perioperative risk factors for postoperative hospital-acquired pneumonia after valve replacement in patients with VHD combined with PH. The established nomogram model has good internal validity and predictive efficacy, which is conducive to the early screening of hospital-acquired pneumonia after valve replacement. Future multi-center studies with large samples are needed to validate the model further.

## Data Availability

The raw data supporting the conclusions of this article will be made available by the authors, without undue reservation.
